# Testing the Effects of a Virtual Reality Game for Aggressive Impulse Management (VR-GAIME): Study Protocol

**DOI:** 10.3389/fpsyt.2019.00083

**Published:** 2019-02-26

**Authors:** Danique Smeijers, Sander L. Koole

**Affiliations:** ^1^Forensic Psychiatric Centre Pompestichting, Nijmegen, Netherlands; ^2^Department of Clinical Psychology, VU University Amsterdam, Amsterdam, Netherlands

**Keywords:** anger management, aggressive behavior, intervention, VR, motivational modification

## Abstract

**Background:** Prior laboratory findings indicate that training avoidance movements to angry faces may lower anger and aggression among healthy participants, especially those high in trait anger. To enrich this training and make it more suitable for clinical applications, it has been developed into a Virtual Reality Game for Aggressive Impulse Management (VR-GAIME).

**Methods:** The proposed study will examine the effects of this training in a randomized controlled trial among forensic psychiatric outpatients with aggression regulation problems (*N* = 60). In addition to the aggression replacement training, participants will play either the VR-GAIME or a control game. Anger will be assessed using self-report. Aggressive impulses will be measured via self-report, a validated laboratory paradigm, and rated by clinicians.

**Discussion:** The authors hypothesize that the combination of the VR-GAIME and regular aggression treatment will be more successful in reducing aggressive behavior. One of the strengths of the proposed study is that it is the first to examine the effects of a motivational intervention in a clinical sample characterized by problems in regulating anger and aggression. Another strength of the proposed study is that the VR-GAIME will be implemented as a multi-session intervention. Additionally, the VR-GAIME applies, for the first time, serious gaming and virtual reality on an avoidance motivation intervention. If positive results are found, the VR-GAIME may be systematically deployed in forensic psychiatric settings.

**Trial registration**: The trial is registered with The Netherlands National Trial Register, number: NTR6986.

## Introduction

Anger and aggression are common to all individuals, however, when it occurs frequently, increases in severity and it disrupts ones functioning, it becomes a significant problem (1). Anger is an acute emotional-physiological reaction that ranges from mild irritation to intense fury and rage (i.e., state anger). The disposition to experience state anger with greater frequency and intensity is referred to as trait anger (2, 3). When anger is not controlled or regulated appropriately, it increases the risk of aggressive behavior (4–6). Aggressive behavior is defined as a destructive behavior directed to another person, object or animal with the intention to cause harm and can be divided into in impulsive (affective, reactive) or instrumental (predatory, proactive) subtype (7, 8).

Aggression in general, and especially the impulsive subtype, is related to poor emotion regulation and poor impulse control (5, 6, 9). Aggressive behavior also has a clear link with antisocial behavior, and is a common basis for referral to forensic psychiatric institutions. Therefore, in treating antisocial and aggressive behavior, much attention has been paid to the importance of emotion regulation and self-control (10–12).

Interventions based on principles of Cognitive-Behavioral Therapy (CBT) have traditionally been the interventions of choice in treating aggression regulation problems (13, 14). These interventions, however, are only partially successful in reducing aggressive behavior and usually only beneficial to a subgroup of individuals (10, 13, 15, 16). Furthermore, CBT interventions appeal to abilities such as reflection, introspection and willingness to genuinely talk about problems in controlling anger and/or aggression. However, these capacities are often limited in individuals with anger or aggression management problems [e.g., (17, 18)]. Additionally, CBT-based interventions target more conscious, deliberate responses, and thus may have little impact on underlying implicit or automatic characteristics (19). When left untreated, these underlying factors might re-emerge during highly provoking and frustrating situations (20). As such, there is room for improvement of traditional aggression regulation interventions.

One new approach focuses on the motivational underpinnings of aggression regulation (21). People high in trait anger tend to have high levels of approach motivation (22), especially in situations when they are socially provoked (21). Which means that, in general, they have an automatic approach tendency toward potential social threat. Furthermore, experiments have shown that blocking approach motivation, for instance, by changing people's posture, can lower state anger and aggression (21). These findings suggest that a motivational approach could contribute to anger and aggression regulation among high-risk populations.

Initial experiments suggest that the motivational approach to anger management can be turned into an intervention (23). These experiments made use of an adapted joystick task that was validated in previous motivational intervention research in the treatment of alcohol abuse and social anxiety (24–26). These studies already proved that such an approach bias modification was successful in reducing alcohol consumption in heavy drinkers and emotional vulnerability in socially anxious individuals. In the experiments by Veenstra et al. (23), healthy participants were asked to perform a task in which they responded to angry or happy faces with a joystick. In the avoidance training condition, participant made avoidance movements to angry faces. In the control condition, participant made approach movements to angry faces. The results showed that after avoidance training, participants reported less angry feelings and expressed less aggressive impulses. These findings were most evident among individuals high in trait anger, who are characterized by the disposition to experience state anger with greater frequency and intensity (2, 3). These results suggest that reducing approach motivation toward social threatening stimuli could be an important addition to conventional anger and aggression regulation interventions.

Although the aforementioned findings are promising, they are limited in important ways. First, the avoidance training of Veenstra et al. (21) consisted of a single session. To increase the long-term effects of the training, its effects should be examined across multiple sessions. Second, the avoidance training was investigated among healthy undergraduate students who were not characterized by severe levels of anger and/or aggressive behavior. To elucidate the clinical relevance of motivational training, its effects among clinical populations need to be investigated. Third and last, the avoidance training of Veenstra et al. (21) used a joystick task that was not very engaging for participants, which could hinder implementation in clinical settings. Especially among individuals receiving treatment for anger and aggression regulation problems, treatment motivation is often lacking (17, 27). To warrant sufficient treatment motivation, it would be desirable to develop a more engaging variant of the motivational training.

For increasing treatment motivation, interventions that use serious gaming and virtual reality technology have been found to be highly effective (28). Serious games refer to games that do not have fun, enjoyment or entertainment as primary purpose but rather training, education or health improvement (29, 30). By introducing playful and interactive elements in an intervention, serious gaming may enhance motivation of the target group (28). Virtual reality (VR), on the other hand, makes use of virtual environments to present digitally recreated real world activities to participants via non-immersive and immersive mediums which can be systematically manipulated to be relevant to patients' problems (31, 32). Another advantage is that VR gives the unique opportunity to investigate and treat underlying behavioral mechanisms in controlled experimental designs that nonetheless possess high ecological validity.

Initial studies of serious gaming and VR in psychiatric treatments have found that these techniques can be used to successfully reduce aggressive behavior, impulsivity, anxiety, posttraumatic stress symptoms and to improve self-regulation and pro-social behavior (33–37). Serious gaming and VR are thus promising tools for enhancing the impact of psychological interventions and have also gained recognition in forensic psychiatry (38). A VR aggression prevention training is even developed and currently examined among forensic psychiatric patients (39).

For the current study, we combined the motivational modification paradigm (21), serious gaming, and VR technology to create a new treatment tool for the treatment of aggressive behavior: the Virtual Reality Game for Aggression Impulsive Management (VR-GAIME). The VR-GAIME is based on the approach-avoidance modification paradigm that was investigated by Veenstraet al. (21). Instead of joystick movements, however, the VR-GAIME manipulates whole body movements in an immersive environment. During the VR-GAIME, each participant gets assigned to the role of a courier who has to collect packages in a shopping street. In the shopping street, the participant is met by avatars who are acting in either an agreeable or disagreeable manner. Agreeable avatars have a happy or neutral facial expression and are saying pleasant things (e.g., “Have a nice day,” “You are wearing such a nice shirt!”). Disagreeable avatars, by contrast, have an angry facial expression and are saying unpleasant things (e.g., What are you looking at? “Get out of my way!”).

The VR-GAIME will be administered during multiple sessions alongside Aggression Replacement Training (ART) among forensic psychiatric outpatients with aggression regulation problems. Patients will be randomly allocated to the VR-GAIME (experimental training condition) or a placebo game (control training condition). In the experimental training condition, patients will be instructed to lean forward (i.e., make an approach movement) in response to agreeable avatars and to lean backwards (i.e., make an avoidance movement) in response to disagreeable avatars. Patients in the experimental training condition will be trained to respond with avoidance behavior to anger-relevant situations. In the control condition, patients will play the same game as patients in the experimental condition. However, patients in the control group will not encounter any disagreeable avatars and will hence not receive any training about anger-relevant situations.

The aim of the current randomized controlled trial is to investigate the effect of the VR-GAIME tool in combination with ART on the level of aggressive behavior of forensic psychiatric outpatients. Anger and aggressive impulses will be measured using self-report and a validated laboratory paradigm as well as by clinician ratings. Also, approach and avoidance behaviors will be assessed using self-report. Additionally, drop-out rates among outpatients receiving aggression treatment are high (40) and is associated with psychopathy and proactive aggression (41, 42). To determine whether drop-out is in line with previous studies or might be less/higher due to the game, we include measures of psychopathy and aggression subtype. Finally, aggression treatment might also change other emotions than anger. To examine this notion a self-report measure for distinct emotions will be included. Also, the effects might be associated with other biases in processing facial expression. To explore this notion, a measure for a hostile interpretation bias will be included. We hypothesize that the combination of the VR-GAIME and ART will be more successful in reducing anger and aggressive behavior relative to the control condition.

## Methods

### Design

The design will be a double blind randomized controlled trial. The trial consists of two conditions: (1) ART and the VR-GAIME; (2) ART and the VR placebo game. Assessments will take place pre-treatment (T1), halfway (T2), and post-treatment (T3). The sample size was calculated for the main research question using G^*^Power software. The sample size was calculated for a two (group: ART and VR-GAIME vs. ART and placebo game) × 3 (assessment: pre vs. halfway vs. post) interaction, with the assumption of a small-to-medium effect size (*eta*^2^ = 0.08) and a power of 1–β = 0.80. This has led to a minimum required sample size of 60.

### Participants and Procedure

Participants will be recruited at “Kairos,” the outpatient unit of Forensic Psychiatric Center the Pomestichting in Nijmegen, The Netherlands. All patients are referred to Kairos because of aggression regulation problems. Admission to Kairos occurs on either obligatory (when sentenced by a judge) or voluntary basis (based on reference by general practitioner which is necessary in secondary care). The antisocial and borderline personality disorder as well as the intermittent explosive disorder are the most common psychopathologies. Inclusion to the study will require to meet the following criteria: (1) male sex; (2) aggression regulation treatment is indicated. Patients will be excluded from the study if they meet the following exclusion criteria: (1) current major depression; (2) current severe addiction; (3) lifetime bipolar disorder; (4) lifetime psychosis. Clinicians at “Kairos” will ask patients who are referred to aggression regulation treatment (group or individual), whether they agree to be contacted about the study. When they agree, patients will be contacted by the researcher. All patients, will receive treatment as indicated whether they participate in the study or not.

After receiving information about the nature of the study, participants will be asked to assign a consent form. Subsequently, patients will be screened by the researchers (who were also trained clinicians in the use of these interviews) with the Structured Clinical Interview for DSM-IV axis II personality disorders [SCID-II; (43)], the Research Criteria set for Intermittent Explosive Disorder [IED-IR; (44)] and, the MINI International Neuropsychiatric Interview for axis I disorders [MINI; (45, 46)] regarding the aforementioned exclusion criteria and in order to confirm diagnosis. Once patients are found suitable for participation, they will proceed with the baseline measurement.

The baseline measurement consists of several questionnaires and two computer tasks. After this pre-treatment assessment (T1), participants will start with their treatment. The patients will be assigned by a computerized random number generator to one of the two conditions: (1) ART and VR-GAIME; (2) ART and VR placebo game. In both conditions, patients are asked to play the game at the outpatient clinic alongside the first five sessions of their treatment. After 5 weeks (T2), after the last VR session, the level of aggressive behavior will be determined by use of a questionnaire. The end of treatment measurement will take place after 12 weeks (T3) and consists of the same questionnaires and computer tasks as the baseline measurement. During the end of treatment measurement (T3), patients will be informed about which condition they participated in. The difference between the two game versions will be explained. In case patients participated in the control condition, the opportunity will be offered to play the experimental game. Patients will be compensated for their participation with a monetary reward (€30,-).

## Intervention

### Aggression Replacement Training (ART)

All patients referred to the “Kairos” because of aggression regulation problems receive the ART (12, 47). The ART is a CBT based intervention. Besides ART for general aggression and violence, ART is also offered for perpetrators of intimate partner violence. This version of the ART is identical to the regular ART except that the partners of the patients are involved during this intervention. Both the regular ART as well as the ART for domestic violence perpetrators consists, as offered by “Kairos,” of two of the three original modules: (1) social skills training and (2) anger control training. Both interventions occur either in groups or individually and consist of two 90-min weekly sessions during 12 weeks. The first 10 weeks consist of the social skills and anger control training. Week 11 consists of a session to integrate all that was learned in the previous weeks. Finally, week 12 consists of an evaluation session. Indication for ART is determined by a multidisciplinary team. The ART therapists (all clinicians at “Kairos,” not involved in the current study as a researcher) are all formerly trained in applying the ART and, in addition, make use of a detailed intervention manual and participate in intervision.

### VR-GAIME

The Virtual Reality (VR) game is based on the underlying principles from the motivational modification paradigm developed by Veenstra et al. (23). Following the philosophy of a “serious game” (28), the training has been developed to be fun and challenging. Participants receive written instructions of what the game entails as well as a demonstration of all actions during the game. During the game, the virtual environment moves automatically as if the participant is actually walking down a shopping street. Participants are able to walk themselves within pre-determined boundaries. To make sure participants can play the game safely, a guardian system will be set up. Due to this guardian system, a virtual laser cage is displayed once a participant cannot walk further in that direction in the real world. This virtual cage ensures that the participant cannot bump into objects in the surroundings, for instance, a wall. To warrant participants' safety, the researcher will be present in the room at all times and will give instructions to reset the participants position if needed.

The VR game has five levels, which are ascending in level of difficulty. During the game, the participant is working as a mail courier. The back-story is that while the courier was driving, he lost several packages. During each level, the participant has to walk down a shopping street in order to collect the lost packages. Once enough packages are collected, the participant can proceed to the next level. In the shopping street, the participant is met by avatars who act in either an agreeable or disagreeable manner. The behavior of the avatars is experimentally manipulated to provide the training component of the game.

Participants will receive the instruction that it is important to respond correctly toward the avatars. Participants in the experimental condition will be instructed to lean forward (i.e., make an approach movement) in response to agreeable avatars and to lean backwards (i.e., make an avoidance movement) in response to disagreeable avatars. In each level, four agreeable and four disagreeable avatars will appear. When an incorrect response is given toward disagreeable avatars, the participants will lose a package.

Instead of the active (experimental) game, half of the participants will play a placebo game. In terms of the general game elements, the placebo game is identical to the original game. The only difference is that, in the placebo game, no disagreeable avatars will appear. As a consequence, avoidance behavior will not be trained in the placebo/control condition. The instruction patients will receive is comparable. This setup was chosen to avoid that patients easily can find out in which condition they participated. Based on prior studies we know that patients tend to discuss such experiences once they participate in group treatment. Participants will be randomly allocated to an experimental or a control condition. Both versions of the game have a maximum duration of 30 min. The two versions of the game are referred to as game 1 and game 2 on the VR computer. Furthermore, the researcher takes position behind the computer screen in order to stay blind for the condition.

Besides the packages and the avatars in the shopping street, the participant will also come across litter on the street. The participant has to make sure he will not walk into this litter otherwise he will lose a package. Thus, when confronted with litter, the participant has to pick up the litter and deposit it behind him. The latter game element is introduced to make the game more varied and engaging.

An additional game element consists of mini-game is an extra challenge and is included to make the game more varied for players. Participants gain entry to the mini-game by collecting bonus packages, which appear as more colorful and somewhat smaller as the regular packages. Once enough bonus packages are collected, the mini-game can be played. During the mini-game, the participants stand underneath a window from which packages are thrown down. Participants need to catch these fallen packages. The higher the level, the more difficult the mini-game will be: The packages will fall faster as well as some rubbish will be thrown out of the window which should not be caught.

## Measures

### Primary Outcome Measure

The *Social Dysfunction and Aggression Scale* [SDAS; (48)] is an observer-scale that measures the severity of state aggressive behavior. It consists of nine items measuring aggression directed to others and two items measuring aggression directed to the self. Items have to be scored on a 4-point scale with 0 = not present and 4 = severely to extremely present as extremes. The SDAS has adequate observer reliability [Cronbach's Alpha =.79; (48)]. In the current study, the SDAS will be rated by the clinician as well as the patient himself. In both bases, aggressive behavior will be rated over a period of 2 weeks. The SDAS will be administered three times: pre-treatment, halfway, and post-treatment (T1, T2, T3). The SDAS as self-report demonstrated acceptable test-retest stability and internal consistency in prior research; intraclass correlation coefficient ranging from 0.651 to 0.82 and Cronbach's Alpha ranging from 0.76 to 0.82 (42).

### Secondary Outcome Measures

Several secondary outcome measures will be included to assess whether the VR-GAIME is associated with aggressive impulses, the tendency to act aggressively, type of aggression (reactive/proactive), state and trait anger, emotional experiences, cognitive bias and approach and avoidance behaviors.

#### Paradigms

The *virtual voodoo doll task* [VVDT; (23)] will be used to measure aggressive impulses. In this task, a picture of a doll is presented on the computer screen. Participants are asked to think about a person who did them wrong. Subsequently, they receive the following information: “In previous research, we have found that you can get rid of negative energy by taking action in response to the person that caused you harm. Imagine that the virtual doll is the person from the situation you just recalled. You now have the opportunity to insert as many pins in the doll at any location you like.”

Participants are able to pick up a pin—presented next to the doll—by left-clicking with their mouse, move it around the screen, and then push the pin into the doll at any place they want. Once a pin is put in the doll it looked as though the pin was stuck in the doll. The position of the pin can be changed as desired. A maximum of 50 pins can be inserted into the doll. The total number of pins each participant will put in the doll will be registered. Furthermore, a screen-capture of the doll will be saved in order to code the position of the pins. The traditional voodoo doll task, with an actual doll and pins, is a reliable measure with convergent and construct validity (49). Also the virtual version of the voodoo doll task is thought to differentiate well between different levels of aggressive impulses, as indicated by putting pins in the vital or less vital body parts [Cronbach's Alpha ranging from 0.88 to 0.91; (23)]. The VVDT will be administered pre- and post-treatment (T1, T3).

The *Hostile Interpretation Bias Task* [HIBT; (50)] will be used to assess a HIB at baseline and end-of-treatment measurement. Photos of faces with emotional affect (angry, fear, disgust, happy) of four male and four female models were selected from the Radboud Faces Database (51). Each affective picture is morphed (using WinMorph 3.01) five times with the neutral image of the same individual, creating 20, 40, 60, 80, and 100% emotion intensity, respectively. The neutral expression is in all models displayed with mouth closed whereas the emotional pictures are displayed with their mouth open. This difference in mouth-opening resulted in pictures showing ambiguous expressions.

The task consisted of a practice block and two experimental blocks. The practice block consisted of 16 trials (8 models × 2 emotions). Only pictures with happy and angry affect and of 100% intensity are used to familiarize participants with the task. Each experimental block consisted of 168 trials (8 models × 4 emotions × 5 intensity levels + 8 neutral images). The order of the pictures is pseudo-randomized and equal in both blocks. Participants are instructed to indicate whether the picture looked hostile or not. When participants believe they see a hostile picture, they have to press the Z-key, otherwise the M-key (on a qwerty keyboard). Participants have to respond as quickly as possible. The picture, size 8.5 cm × 10.5 cm, is presented for 4 s, in the center of the computer screen, against a black background. The pictures remain on the screen until a response is given or until 4 s had passed. After a pretrial pause of 1 s, a new picture is displayed immediately.

Labels are displayed in the left (Yes, hostile) and right (No, not hostile) bottom corner of the screen in white Arial font, size 30. Responses given by pressing the Z-key, indicating that the participant saw a hostile picture, are defined as “hostile” responses. If a response is not given within 4 s, the words “Too late” appears on the screen in red. A hostile interpretation bias is defined as the percentage of “hostile” responses to the emotional pictures. The hostile responses are pacifier coded (0 = no, not hostile, 1 = yes, hostile), and the mean is calculated which indexes the percentage of the pictures that were interpreted as hostile. Trials without a response (due to late responding) are not taken into account. The HIBT has good test-retest reliability, except for happy faces [*r* ranging from 0.774 to 0.908; happy faces *r* =.295; (50)]. The HIBT will be administered pre- and post-treatment (T1, T3).

#### Questionnaires

The *Behavioral Inhibition System/Behavioral Activation System* [BIS/BAS; (52)] scale is a well-validated scale contains 20 items measuring individual differences in personality dimensions that reflect the sensitivity of two motivational systems: the aversive (BAS) and the appetitive (BIS) system. The BIS subscale consists of seven items whereas the BAS subscale consists of 13 items. The BAS subscale is subdivided in three subscales: fun seeking (4 items), reward responsiveness (5 items), and drive (4 items). Participant rate how true the statements are for them on a 4-point Likert scale (0 = very true, 4 = very false). Prior research has shown that the Dutch translation exhibits adequate internal consistency [Cronbach's alphas ranging from 0.59 to 0.79; (53)]. The BIS/BAS will be administered at pre- and post-treatment (T1, T3).

The *Reactive Proactive Questionnaire* [RPQ; (54, 55)] is a 23-item self-report questionnaire to measure reactive and proactive aggression. The reactive subscale consists of 11 items whereas the proactive subscale consists of 12 items. The items are rated 0 (never), 1 (sometimes), or 2 (often). Prior research indicates that the Dutch translation has good internal consistency (Cronbach's Alpha = 0.91) and adequate convergent (all *r* < 0.16), criterion (delinquents from prison and forensic mental health scored higher than non-offenders) and construct validity [violent offenders show more proactive aggression than non-offenders, *p* < 0.001; (54)]. The RPQ will be administered at pre- and post-treatment (T1, T3).

The *Aggression Questionnaire* [AQ; (56)] is a self-report questionnaire to assess an overall trait of aggression. It consists of 29 items that are divided into four subscales: physical aggression, verbal aggression, anger, and hostility. The items are scored on a 5-point Likert scale (1 = extremely unlike me to 5 = extremely like me). Prior research indicates that the Dutch translation has adequate psychometric properties [Cronbach's Alpha = 0.86; (57)]. The AQ will be administered pre- and post-treatment (T1, T3).

The *Self-Report Psychopathy Short-Form* [SRP-SF; (58, 59)] is a self-report measure of adult psychopathic features. The SRP-SF consists of 29 statements that are divided into four subscales: Interpersonal manipulation, callous affect, erratic life styles, and criminal tendencies. Participants have to rate the extent to which they agree with these statements on a 5-point Likert scale (1 = disagree strongly, 5 = agree strongly). Prior research indicates that the Dutch version of the SRP-SF has good internal-consistency and test-retest reliability [Cronbach's Alpha ranging from 0.58 to 0.73 and *r* ranging from 0.60 to 0.86; (60)]. The SRP-SF will only be administered pre-treatment (T1, T3).

The *State Trait Anger Scale* [STAS; (61)] has been designed to measure state and trait anger. It is a self-report questionnaire of 20 items subdivided in two subscales: state and trait anger. State anger refers to an emotional condition of a patient, which is consciously experienced and fluctuates over time. Trait anger refers to a stable personality quality: the disposition to become angry, a tendency that differs much among individuals. The STAS will be administered pre- and post-treatment (T1, T3). Prior research indicates that the Dutch translation has proven psychometric properties [Cronbach's Alpha = 0.91 for trait aggression and.95 for state aggression; (62, 63)].

The *Discrete Emotions Questionnaire* [DEQ; (64)] is a self-report measure of the following distinct state emotions: fear, anxiety, sadness, anger, disgust, happiness, relaxation, and desire. The participants are asked to think of someone with whom they have many conflicts. Subsequently, they are asked to indicate to what extent they experience these emotions regarding this person on a 7-point Likert scale (1 = not at all-−7 = an extreme amount). Thirty-six emotions are listed which are all synonyms for the eight aforementioned emotions. The original version has good internal consistency [Cronbach's Alpha ranging from 0.95 to 0.97; (64)]. The DEQ will be administered pre-treatment, half-way and post-treatment (T1, T2, T3) in order to assess changes in state emotions during the intervention.

### Statistical Analyses

This RCT has a repeated-measures design with both between- and within-subject variables. The main between-subject factor is experimental condition: ART + VR-GAIME vs. ART + VR placebo game. The main within-subject factor is assessment time: aggressive behavior at pre-intervention (T1), halfway (T2), and post-intervention (T3). To examine whether aggressive behavior will change over time, a linear mixed model will be used (SPSS, version 25). One advantage of this analysis is that it is possible to include individuals with incomplete data, without imputing data (65). This method was favored because there is a plausible chance that the halfway and/or end-of-treatment measurements will not be completed by all participants, given that dropout rates in forensic psychiatric settings are usually high.

### Main Effects of Training

The basic model will be a repeated-measures design with aggressive behavior as measured with the patient-rated SDAS as dependent variable and Time of measurement (baseline, half-way, end of treatment) as within-subjects factor and Training (VR-GAIME vs. placebo) as between-subjects factor. Repeated covariance type will be set at diagonal, which assumes heterogeneous variances and no correlation between elements (65). With respect to Time, the slope will be set as a fixed effect and the intercept as a random effect. This random effect is defined in order to assess variation in the dependent variable because variation among individuals, regarding change in aggression over time, was assumed (66, 67). The covariance type for the random effects will be set at unstructured as a completely general covariance matrix (65).

Second, a similar linear mixed model will be run, now with the SDAS rated by the clinician as dependent variable and Time of measurement (baseline, half-way, end of treatment) as within-subjects factor and Training (VR-GAIME vs. placebo) as between-subjects factor, to examine whether aggressive behavior decreased during treatment according to clinicians. Another similar linear mixed model will be conducted with the DEQ as dependent variable and Time of measurement (baseline, half-way, end of treatment) as within-subjects factor and Group (VR-GAIME vs. placebo) as between-subjects factor, to examine whether distinct emotional experiences change during treatment.

Third, it will be investigated whether the secondary outcome measures, which related to trait aggression, trait anger, reactive and proactive aggression, aggressive impulses, behavioral inhibition and disinhibition and hostile interpretation bias, change after treatment, and whether this change is different among the two training conditions. A linear mixed model can only analyze one dependent variable. As the current sample size is relatively small, a large amount of linear mixed models cannot be executed. Therefore, a different statistical approach is chosen: difference scores for all secondary outcome measures will be calculated; the baseline score will be subtracted from the end of treatment score. Negative scores indicate a reduction during treatment whereas positive scores indicate an increase during treatment. Subsequently, a MANOVA will be conducted to explore whether change in secondary outcome measures during treatment is different between the two training conditions.

### Individual Differences in Treatment Effects

To explore individual differences in the effects of Treatment, we will analyze as potential moderators trait aggression, trait anger, reactive and proactive aggression, aggressive impulses, psychopathy, behavioral inhibition and disinhibition, and hostile interpretation bias measured at baseline. We will examine these moderators by adding main effects of these variables and two-way interactions of the measures and Time/Training and the three-way interaction between potential moderator, Time, and Training to the basic linear mixed model, with SDAS as self-report, as described above. To be able to interpret the results all independent variables will be centered; the sample mean will be subtracted from the individual participant's mean. We will run these analyses only for the patient-rated SDAS.

## Discussion

One of the strengths of the proposed study is that it is the first to examine the effect of a motivational intervention in a clinical sample characterized by problems in regulating anger and aggression. Previous studies on the effects of avoidance training on anger and aggressive impulses consisted of a single session joystick training among undergraduate students (23). The automatic approach of social threat displayed by individuals high in trait anger (21) might be more persistent among forensic psychiatric patients typified by higher levels of trait anger and problems with aggression regulation. In this regard, a single session might not be sufficient. Another strength of the proposed study is that the VR-GAIME will be implemented as a multi-session intervention. This also provides the unique opportunity to investigate motivational tendencies over a longer period of time. The proposed study will, therefore, have important theoretical implications, by providing a further test of motivational models of anger management (68).

Additionally, the proposed study makes use of innovative technologies. The VR-GAIME applies, for the first time, serious gaming and virtual reality on an avoidance motivation intervention. These techniques require more patient engagement. Furthermore, automatic processes are trained in more life-like situations which likely increases the ecological validity of the intervention. Another advantage of these techniques is that it provides an opportunity to develop a training tool that patients like to use. Treatment dropout in forensic psychiatric settings is extremely high (40). This might be due to high psychopathic traits and proactive aggression, but also the low levels of treatment motivation may play a role. It is conceivable that, once patients enjoy playing the game, they tend to dropout less end may benefit more from treatment.

One of the limitations of the proposed study is that, even though the control game does not train avoidance behavior, it does train approach behavior toward agreeable avatars. It may be that the control condition also will have positive effects. Additionally, patients will participate voluntarily, which means that it is possible that the most severe patients may not be included as they are not willing or capable to participate. This could introduce a selection bias that might limit the generalizability of the results.

The innovative approach chosen in this study will hopefully have added value to traditional cognitive anger management interventions and contribute to the decrease of disproportionate aggressive behavior. When positive results are found, the VR-GAIME may be systematically deployed in forensic psychiatric settings.

## Ethics Statement

The present study was reviewed by the Medical Ethical Committee (METc) of the Vrije Universiteit (VU) medical center. The committee judged that the Medical Research Involving Human Subjects Act (WMO) did not apply to this study (2017.563). Moreover, the study was approved by the local ethics committee of the Faculty of Social Sciences at the Radboud University in Nijmegen (ECSW2017-1303-499).

The proposed study will provide a further test of embodied motivational models of anger and aggression management and of VR-techniques that complement traditional cognitively based interventions. The VR-GAIME is intended to provide a training for an implicit and automatic mechanism which is enjoyable for patients as well. The results of the proposed study will be reported in future conference presentations and peer-reviewed journals.

## Author Contributions

DS wrote the first draft of the present manuscript. SK contributed to final manuscript editing. DS and SK contributed to the development of the VR-GAIME.

### Conflict of Interest Statement

The authors declare that the research was conducted in the absence of any commercial or financial relationships that could be construed as a potential conflict of interest.
